# In Vivo Regenerative Potential of *Coprinus comatus* in Pancreatic Tissue After Acute Stress with Chronic Consequences

**DOI:** 10.3390/molecules30112261

**Published:** 2025-05-22

**Authors:** Nebojša Stilinović, Ana Tomas, Saša Vukmirović, Nebojša Kladar, Miloš Čanković, Maja Đanić, Michał Seweryn Karbownik, Aleksandar Rašković, Ivan Čapo

**Affiliations:** 1 Department of Pharmacology, Toxicology and Clinical Pharmacology, Faculty of Medicine, University of Novi Sad, 21000 Novi Sad, Serbia; ana.tomas@mf.uns.ac.rs (A.T.); sasa.vukmirovic@mf.uns.ac.rs (S.V.); maja.djanic@mf.uns.ac.rs (M.Đ.); aleksandar.raskovic@mf.uns.ac.rs (A.R.); 2 Department of Pharmacy, Faculty of Medicine, University of Novi Sad, 21000 Novi Sad, Serbia; nebojsa.kladar@mf.uns.ac.rs; 3 Department of Dental Medicine, Faculty of Medicine, University of Novi Sad, 21000 Novi Sad, Serbia; milos.cankovic@mf.uns.ac.rs; 4 Department of Pharmacology and Toxicology, Medical University of Lodz, 90-752 Lodz, Poland; michal.karbownik@umed.lodz.pl; 5 Department of Histology, Faculty of Medicine, University of Novi Sad, 21000 Novi Sad, Serbia; ivan.capo@mf.uns.ac.rs

**Keywords:** pancreas plasticity, alloxan-induced diabetes, molecular docking analysis, morphometric study

## Abstract

The edible mushroom *Coprinus comatus* has a long history of use in metabolic diseases, which is increasingly documented by modern research. Due to its favorable nutritional composition, it was assumed that this mushroom could accelerate tissue recovery after acutely induced damage with subsequent disturbance of primarily carbohydrate metabolism. To test this hypothesis, the alloxan diabetes model was used, where experimental animals’ change in body weight and biochemical and histological indicators of recovery were monitored. Before performing the in vivo part, HPLC analysis of the *C. comatus* extract was carried out with subsequent in silico and in vitro tests. Comparing the animals treated with the mushroom in three different doses, no significant change in body weight was observed. Still, the change was also noticed in the lipid status and glycemia, with a dose-dependent beneficial effect. Morphometric analysis of pancreatic tissue stained by immuno-histochemical methods showed that long-term treatment with *C. comatus* leads to increased numerical density, nuclear volume, and absolute number of beta cells of the islets of Langerhans, which suffered severe damage after alloxan administration. Overall, *C. comatus* may contribute to faster tissue recovery after acute diabetic-relevant damage with chronic consequences.

## 1. Introduction

*Coprinus comatus* (O.F. Mull.) Pers. Agaricaceae is a mushroom whose pharmacological effects have been the subject of many preclinical studies in recent decades. Among the investigated pharmacological effects are hepatoprotective [1,2], nephroprotective [3], antimicrobial [4,5], and antiobesity effects [6], but the most common is the antidiabetic effect [4,7,8,9], which may stem from the antioxidant effect [10,11]. One of the reasons for the beneficial effect of this mushroom in metabolic diseases, such as diabetes, where lipid disorders also occur, may be its favorable nutritional composition in addition to carbohydrate metabolism disorders. *C. comatus* has an extremely high content of dietary fibers, proteins, and oligo-elements (selenium, zinc, and copper) and a low content of fats and simple sugars. At the same time, some samples also contain different forms of vitamin E. The fats found in the mushroom are mostly polyunsaturated, with a relatively high presence of omega-3 fatty acids [2,4,5,10,12]. In addition, many phenolic compounds have been identified in the *C. comatus* mushroom, primarily powerful antioxidants, and some of them are considered to have antidiabetic effects [11,13,14]. It is interesting to note that in the studies that use the cultivated mushroom *C. comatus*, it is concluded that there is no difference in the nutritional and pharmacological properties compared to those of mushroom samples collected from the wild. In addition, in some experiments, a more substantial effect of the cultivated mushroom is observed [2,5,10]. Looking comprehensively at the results of the mentioned research, it is assumed that *C. comatus* can help in the regeneration of tissue that is exposed to both acute and chronic stress [2,4].

As already stated, the antidiabetic effect is the most-often-attributed positive effect of the mushroom *C. comatus*, and on the other hand, alloxan is a substance that is still very frequently used to induce experimental diabetes [7,9,15,16]. Alloxan’s mechanism of toxicity primarily involves the generation of reactive oxygen species that target pancreatic beta cells selectively. Still, it has also been shown to damage liver and kidney tissue to a certain degree. At first, alloxan-induced diabetes is very similar to human type 1 because it leads to selective damage to the insulin-producing cells of the islets of Langerhans. However, it is not entirely the same because after a certain time, recovery occurs [15,16]. Preclinical research has been showing that pretreatment with certain substances can alleviate the toxicity of alloxan, presumably by increasing the body’s antioxidant capacity [15,17]. It is also possible that the damage caused by oxidative stress and inflammation in the pancreatic tissue can be repaired by the use of substances with antioxidant, anti-inflammatory, and regenerative potential [4,18].

In previous years, there has been an increase in studies dealing with the plasticity of the pancreas as a mechanism of adaptation to stress. These studies point out that the recovery of the endocrine pancreas occurs when individual cells enter the trans-differentiation process, so alpha, gamma, and delta cells take on the characteristics of beta cells and begin to secrete insulin [19,20,21,22]. In addition to ELISA, RNA sequencing, and other biochemical and molecular methods, the process of trans-differentiation, i.e., maturation of insulin-producing cells, was observed by immunohistochemical analysis, which helps in locating cell migration and the formation of new cell sets [13,15,18,21]. Finally, it should not be ignored that in experimental diabetes, the remaining beta cells also undergo a replication process, thus trying to restore endocrine function. However, the tissue of the endocrine pancreas does not belong to highly regenerative tissues, such as epithelial ones, so beta cells need more time for maturation and favorable conditions to repair damage [20,21,23].

Considering the peculiarity of alloxan diabetes, it represents an appropriate experimental model for examining the regeneration of the pancreas after acute stress with chronic consequences. The antioxidant and regenerative potential of the *C. comatus* mushroom has been demonstrated in several preclinical studies, including our previous ones. Thus, our goal was to further examine the above-mentioned positive effects in a study on Wistar rats with alloxan diabetes, with an emphasis on the morphometric analysis of pancreatic tissue and to link it with biochemical parameters of recovery.

## 2. Results

The results of the HPLC-DAD analysis, employed to check the quantity of phenolic compounds in the sample of *Coprinus comatus* mushroom, are presented in Table 1. HPLC analysis of the aqueous extract of *C. comatus* showed the presence of chlorogenic acid as a principal phenolic constituent. Besides chlorogenic acid, caffeic acid and p-Coumaric acid were also identified, while the other investigated phenolics presented in Table 1 were under the limit of detection (chromatograms are posted as Supplementary Materials S1).

We have conducted a molecular docking analysis to compare the binding affinities of ligands with reference compounds. The results are presented in Table 2. The binding energies of chlorogenic acid in complexes with alpha-glucosidase are almost equal to the binding energy of the reference compound (acarbose). Chlorogenic acid also has the highest negative value, compared to other ligands, in complexes with DPP4 and alpha-amylase. However, these affinities are lower in comparison to the reference compounds. The highest affinity of chlorogenic acid may be partly explained by its chemical structure, including the largest number of H bonds and its share in the overall docking score. The second-ranked ligand is caffeic acid with 3 OH, followed by p-coumaric and cinnamic acid with two and one hydroxyl groups, respectively. Slightly different results were observed in the case of DPP4, where p-coumaric acid ranked in second place, indicating that other interactions, in addition to H bonds, contribute to the overall docking score.

After in silico analysis, we performed the in vitro analysis of *C. comatus* extract (Table 3). By comparing the results obtained, it is evident that the aqueous extract of the mushroom *C. comatus* exhibits the least significant impact on α-amylase, with an (50% enzyme inhibition) IC_50_ value of 13,629.74 µg/mL. The IC_50_ values for α-glucosidase and DPP-4 were 757.48 µg/mL and 1650.3075 µg/mL, respectively. Still, the inhibitory effects of *C. comatus* extract on all enzymes tested here are much lower than those of the positive controls, acarbose and sitagliptin. Although chlorogenic acid had a binding affinity for the investigated enzymes similar to sitagliptin/acarbose, the chlorogenic acid content in the mushroom extract was low, which might explain the lower inhibitory effects of *C. comatus* extract.

According to the body weight measurement results, 42-day treatment with *C. comatus* in all three studied doses is body-weight-neutral compared with same-length saline treatment in nondiabetic and diabetic animals (Table 4). Namely, at the end of the 42-day experiment, all groups of animals had body weight gain. Although the lowest body weight gain was in the control group of diabetic rats (42.67 ± 8.48), this change was statistically insignificant in comparison with other diabetic groups (F (3, 20) = 2.066, *p* = 0.137) because of high intragroup variations. The change in body weight of healthy animals was similar to that of diabetic animals, regardless of the treatment. Namely, although the control group of animals (71.17 ± 8.35) had the lowest increase in body weight, that change did not achieve statistical significance.

**Figure 1:**
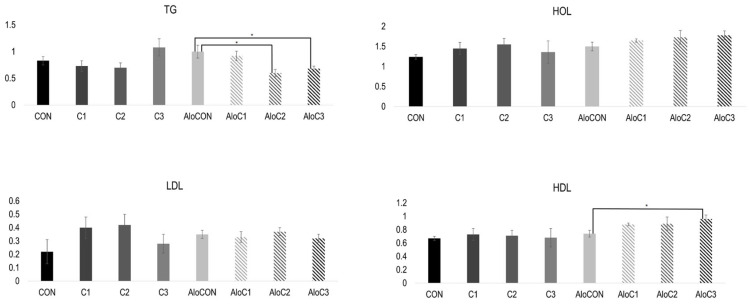
Lipid status of animals treated for 42 days with *C. comatus* or saline (mean ± SEM; n = 6; TG—total triglycerides (mmol/L); HOL—total cholesterol (mmol/L); HDL—high-density lipoprotein cholesterol (mmol/L); LDL—low-density lipoprotein cholesterol (mmol/L); *—*p* < 0.05).

The results of the lipid status are presented in Figure 1. A Kruskal–Wallis test revealed a statistically significant difference in the TG values for the diabetic animal groups, *X*^2^ (3, n = 24) = 10.439, *p* = 0.015. Post hoc comparisons using the Mann–Whitney U Test indicated a significant difference between the AloCON and AloC2 groups (U = 2.000, z = −2.562, *p* = 0.009) and between AloCON and AloC3 (U = 4.000, z = −2.342, *p* = 0.016). The Bonferroni adjustment was used with an alpha level of 0.05/3 = 0.017 to determine the significance of AloCON and other groups of diabetic animals. There was a statistically significant difference at the *p* < 0.05 level in the HDL values for the diabetic animal groups (F (3, 20) = 5.242, *p* = 0.008).

At the beginning of the experiment, there were no differences in glycemia values (BG start) between groups of animals. However, there were changes at the end (BG end) of the 42-day treatment with the mushroom, so in the group of animals that received *C. comatus* in a dose of 1.67 g/kg (C2), a statistically significantly lower glycemic value was recorded compared to all other tested groups (F (3, 20) = 8.127, *p* = 0.001). Furthermore, the change in the glucose value after the oral glucose tolerance test (ΔBG) was not significant compared to that in the healthy animals (Table 5).

The blood glucose values of the groups of animals before the application of alloxan (BG before) were not statistically significant, and comparing the experimental groups to each other, no change was observed even 48 h after the application of alloxan (BG alo). However, there was a statistically significant difference at the *p* < 0.05 level in the blood glucose end-of-treatment (BG end) values for the diabetic animal groups (F (3, 20) = 5.296, *p* = 0.007). Additionally, there was a statistically significant difference at the *p* < 0.05 level (Table 6) in the blood glucose change (ΔBG) values for the diabetic animal groups (F (3, 20) = 3.674, *p* = 0.029). In both cases, the group treated with *C. comatus* at the highest dose (AloC3) had significantly different values than those treated with saline (AloCON). Furthermore, bivariate Pearson’s test showed a statistically significant correlation between the *C. comatus* dose and BG end, BG AUC, or ΔBG values of diabetic animals at the *p* < 0.01 level.

Pearson’s correlation coefficient of −0.635 shows a strong negative correlation between BG end and *C. comatus* dose. A moderate negative correlation was evident for BG AUC with a Pearson coefficient of −0.527. Finally, for ΔBG, the coefficient was 0.578, which is a moderate positive correlation.

**Figure 2:**
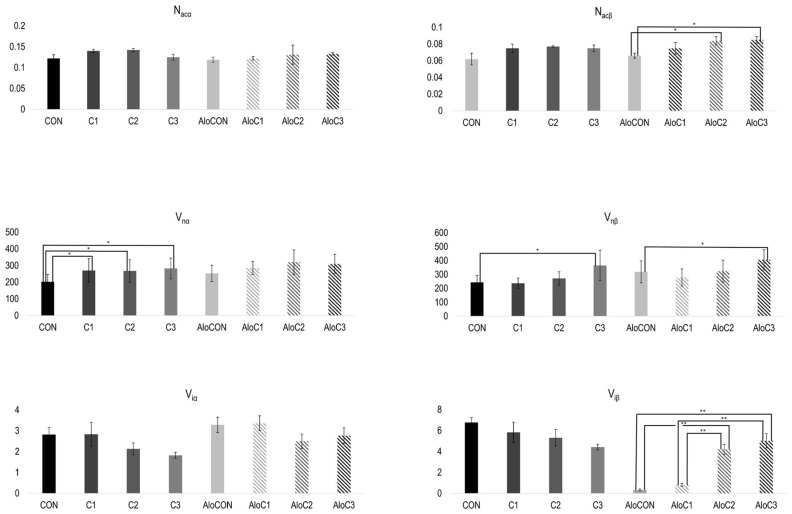
Morphometric analysis of numerical density (N_ac_; 10^−2^), average volume (V_i_; 10^6^), and corrected nuclear volume (V_n_; 10^6^) of α and β cells (mean ± SEM; n = 6; *—*p* < 0.05; **—*p* < 0.01).

In Figure 2, the results of the morphometric analysis of pancreas samples are presented. The numerical density of alpha cells (N_acα_) was without differences between the examined groups of animals, both in healthy and diabetic ones. On the contrary, significantly higher values for the numerical density of beta cells (N_acβ_) were recorded in diabetic animals treated with the middle and highest doses compared to the that in the AloCON group (F (3, 20) = 3.617, *p* = 0.031). Moreover, linear trend analysis revealed a positive relationship between the *C. comatus* dose and the numerical density of beta cells (Figure 2, *p* = 0.005). A similar pattern was observed for the average volume values of alpha (V_iα_) and beta (V_iβ_) islet fractions. There were no differences in the alpha fraction. At the same time, in beta, there was a statistically significantly higher volume value in the diabetic groups that received the medium and highest doses of *C. comatus* compared to the group treated with saline solution and the group with the lowest dose of the mushroom (F (3, 20) = 30.542, *p* < 0.001). Again, trend analysis indicated a positive relationship between the *C. comatus* dose and average volume values of the beta islet fraction (Figure 2, *p* < 0.001). Observing the nuclei of alpha and beta cells, it was evident that the average value of the corrected nuclear volume of alpha cells (V_nα_) of healthy animals in all three groups that received mushrooms was significantly higher compared to that of the control group (F (3, 20) = 15.697, *p* < 0.001). The same was observed in animals with diabetes, but without achieving statistical significance of the difference. The mean value of corrected beta cell nuclear volume (V_nβ_), in both healthy and diabetic animals, was statistically significantly higher in the group treated with the highest dose of mushroom compared to the respective controls, CON (F (3, 20) = 15.697, *p* < 0.001) and AloCON (F (3, 20) = 30.542, *p* < 0.001).

**Figure 3:**
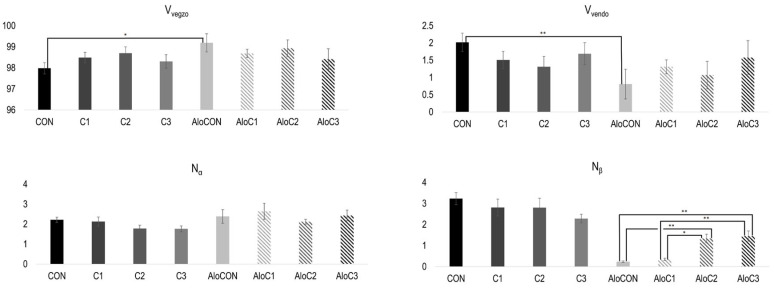
Morphometric analysis of volume density of exocrine (V_vegzo_; %) and endocrine (V_vendo_; %) parts, and number of α (N_α_; 10^3^) and β (N_β_; 10^3^) cells per islet (mean ± SEM; n = 6; *—*p* < 0.05; **—*p* < 0.01).

The rest of the results of the morphometric analysis are presented in Figure 3. The difference in the volume densities of the exocrine (V_vegzo_) and endocrine (V_vendo_) parts is observed only when comparing the controls of healthy (CON) and diabetic (AloCON) animals to each other, where it can be seen that the saline-treated healthy animals had a significantly bigger endocrine part (t = ±3.453, *p* = 0.016). Finally, calculating the average values of the number of alpha and beta cells per islet, it can be seen that treatment with mushrooms in the medium and highest doses led to a statistically significant increase in the number of beta cells (N_β_) in diabetic animals compared to the respective control and the group with the lowest dose of *C. comatus* (F (3, 20) = 15.697, *p* < 0.001). Furthermore, linear trend analysis showed a positive relationship between the *C. comatus* dose and the number of beta cells (Figure 3, *p* < 0.001).

**Figure 4:**
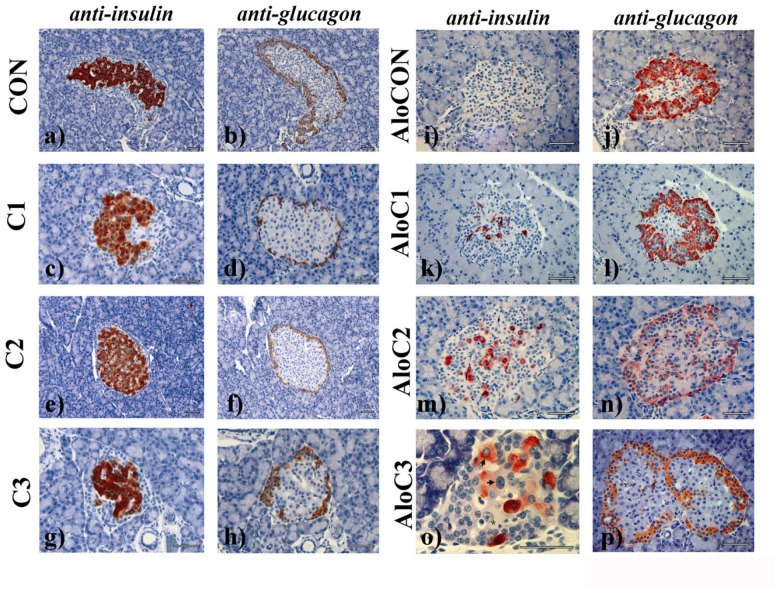
Immunohistochemical analysis of normoglycemic and diabetic (Alo) rats’ Langerhans islets after 42 days of treatment with *C. comatus* in three different doses (C1—0.835; C2—1.67; C3—3.34) or saline (CON); the scale bar represents 50 nm.

Application of the mushroom alone (Figure 4c–h; C1–C3 groups) did not change the cytoarchitectonics of the insulin (central) and glucagon (peripheral) cell compartments, which was the case for the control group (Figure 4a,b; CON group) of animals as well. The islets in the AloCON group were, for the most part, cytoarchitectonically disturbed with the loss of the insulin compartment and a remnant of connective vascular tissue inside of it. In contrast, the glucagon cells along the “jagged” border with the exocrine part remained concentrated in multiple layers on the islets’ periphery (Figure 4i,j). A similar pattern was evident in the group treated with the lowest dose of mushroom (Figure 4k,l). In groups AloC2 and AloC3, after 42 days of the mushroom administration, a marked regeneration of the insulin compartment could be observed, which was characterized by the existence of large cells, a large nucleus, and a prominent nucleolus, the cytoplasm of which was either slightly (asterisk) or significantly positive (arrow) for insulin (Figure 4m,o). Many islets were characterized by almost complete restitution of the endocrine–exocrine border with a diffuse distribution of glucagon cells (also in the center of islets), a clear sign of a regenerative islet (Figure 4n,p).

## 3. Discussion

In the past, plants were commonly believed to be the leading suppliers of phenolic compounds. However, recent discoveries have shown that mushrooms also contain a significant amount of phenolics, as supported by multiple studies that highlight the potent antioxidant properties of both cultivated and wild-growing mushrooms [2,4,11,13]. The antioxidant capacity of mushrooms closely aligns with their total phenolic content, suggesting that phenolics may be the primary contributors to the antioxidant activity observed in edible mushrooms. On the other hand, numerous studies have documented in vitro antidiabetic activity of the same phenolic compounds, expressed as α-amylase, α-glucosidase, and dipeptidyl peptidase-4 enzyme inhibitory activity [8,14,24,25]. In the present study, HPLC-DAD analysis of the aqueous extract of *C. comatus* identified chlorogenic acid as a principal phenolic constituent and detected the presence of caffeic acid and p-Coumaric acid. This is in agreement with the results of Tešanović et al., who found chlorogenic acid as one of the main constituents of *C. comatus* fruiting body water extract [11]. Our previous studies of the *C. comatus* phenolic profile identified p-Coumaric acid and caffeic acid in pure methanolic and 70% methanolic extracts, which are still in much lower quantities. Additionally, in those studies, by scanning mass spectra, we did not find chlorogenic acid, which was not the case for the water extract analyzed here [2,10].

In silico methods effectively reduce the need for extensive high-throughput screenings. Molecular docking is a widely employed in silico method in structure-based drug design due to its remarkable capability to accurately predict the conformation of small-molecule ligands within the designated target binding site [14,24,25,26]. A molecular docking analysis showed that chlorogenic acid has the highest affinity to all the studied proteins, with the most pronounced effect in the case of alpha-glucosidase, where the affinity of chlorogenic acid is almost equal to that of the reference compound. Recent research has revealed that polyphenols possess stable chemical characteristics that effectively inhibit the enzymes α-glucosidase, α-amylase, and DPP4. It is particularly intriguing because polyphenol compounds offer a vast array of potential inhibitors, making them valuable resources for the development of functional foods and nutraceuticals aimed at treating diabetes mellitus [24,25,27]. Recently, a group of authors from Spain demonstrated that phenolic acids are involved in modulating the activity of sugar digestion enzymes and that the inhibitory effect depends on the chemical structure of phenolics, more precisely, the number of hydroxyl groups [24]. Furthermore, an in silico molecular docking study of 14 phenolics from Qingke barley fresh noodles showed that chlorogenic acid and quercetin had better DPP-4 inhibitory effects than other compounds. It was demonstrated that chlorogenic acid and quercetin formed hydrogen and hydrophobic bonds in the active pocket area, which affected DPP-4 conformation and inactivated its enzymatic activity [27].

After in silico analysis, we performed the in vitro analysis of *C. comatus* extract and found that the inhibitory effects of *C. comatus* extract on all enzymes tested were much lower than those of the positive controls, acarbose and sitagliptin. Although chlorogenic acid had a binding affinity for the investigated enzymes similar to that of sitagliptin/acarbose, the chlorogenic acid content in the mushroom extract was low, which might explain the lower inhibitory effects of *C. comatus* extract. However, one of the in vitro studies revealed the inhibitory effects of crude polysaccharides extracted from *C. comatus* on α-amylase activity. The authors found that with increased intracellular crude polysaccharide concentrations in extract, its inhibitory effect on α-amylase also rises [28]. Moreover, the inhibitory effect of *C. comatus* methanolic extract was evaluated in the comparative analysis with five other edible and medicinal mushrooms: *Agaricus blazei*, *Cordyceps militaris*, *Inonotus obliquus*, *Morchella conica*, and *Phellinus linteus*. The analysis of enzyme inhibition revealed that *C. comatus* had the lowest IC_50_ value, approximately 714.45 µg/mL. In contrast, no inhibition of α-amylase was observed at the tested concentration with methanolic extracts of *Morchella conica* and *Cordyceps militaris*. In the case of α-glucosidase inhibition, *Inonotus obliquus* methanolic extract exhibited the most promising potential (220.31 µg/mL), and *C. comatus* achieved IC_50_ of 322.74 µg/mL, which is more than double compared to our result [29]. Studies that focused on organic solvent extracts from various medicinal and edible mushrooms, such as methanol and hexane, demonstrated significantly higher inhibition of this enzyme than the current research. For certain mushroom species, including *Chroogomphus rutilus*, *Leucopaxillus gentianeus*, and *Amanita ovoidea*, the IC_50_ values for α-amylase were less than 100 µg/mL. Regarding α-glucosidase, the same study reported that *Chroogomphus rutilus*, *Leucopaxillus gentianeus*, and *Amanita ovoidea* mushroom extracts with organic solvents exhibited concentrations of around 500 µg/mL for 50% inhibition of this enzyme [30]. Furthermore, recent studies have highlighted various natural bioactive compounds, including alkaloids, flavonoids, terpenoids, and phenols, as well as plant extracts commonly used in traditional medicine such as green/white tea, citrus, berries, rosemary, and grapes, all of which have demonstrated DPP-4 inhibitory effects [27,31]. In addition, the previously mentioned study tested the effect of phenolics identified in Qingke barley fresh noodles on DPP-4 in vitro activity. In vitro enzyme activity inhibition assay showed that among the 14 phenolic compounds, chlorogenic acid had the lowest IC_50_ value, still more than a thousand times higher than that of the positive control, sitagliptin [27]. Nevertheless, certain phenolic compounds have the potential to exhibit significant inhibitory activity against the DPP-4 enzyme, and one of them is naringin, abundantly present in the peels of orange. A virtual docking study revealed tight binding of naringin to DPP-4, while an in vitro inhibition assay showed better activity of naringin than sitagliptin [14]. Therefore, according to the results of our previous study, we expected that water as a solvent would contribute to a better extraction of phenolic compounds because in that study, by adding water to methanol, we obtained a higher concentration of phenolic compounds [2]. We further hypothesized that a higher content of phenolic compounds would significantly inhibit the enzymes responsible for carbohydrate metabolism in in vitro conditions, but this was not the case.

The present study found that a 42-day treatment with *C. comatus* does not affect body weight in nondiabetic and diabetic rats. In our study, the change in body weight of diabetic animals was similar to that of healthy animals, regardless of the treatment. Namely, although the diabetic control group of animals (AloCON) had the lowest increase in body weight, that change did not achieve statistical significance. In our previous study with chronic *C. comatus* treatment (42 days) of Wistar rats, there was no statistically significant difference in body weight change between control (saline) and mushroom-treated groups [2]. On the contrary, our study with 7-day administration of *C. comatus* found that such treatment resulted in a significantly lower increase in body weight compared to that in the control (saline) group [9]. Likewise, *C. comatus* extract demonstrated the ability to prevent the increase in fat mass and body weight in Sprague–Dawley rats fed a high-fat diet. Following a 5-week treatment with *C. comatus*, a significant reduction in body weight was observed in that study [6]. Additionally, other researchers pointed out that *C. comatus* significantly impacted the body mass of experimental mice. In a study conducted by Han and Liu, mice treated with *C. comatus* for ten days following alloxan administration exhibited a statistically significant increase in body weight compared to mice that received only saline [31]. Furthermore, Yu et al. demonstrated that the application of polysaccharides from *C. comatus* led to an increase in body mass among diabetic mice, in contrast to those that did not receive treatment, where such an effect was not observed [27]. Finally, in a study that examined whether there is a spontaneous recovery in animals exposed to alloxan, it was observed that two weeks after the application of alloxan, animals developed long-term diabetes, with differences in the body weight of healthy and diabetic animals, which was not evident in our study [15]. It is worth mentioning that all previously described studies differ in methodology, duration, or species from the present experiment. Thus, it is difficult to compare their results with ours.

We have found a statistically significant difference in the TG and HDL values for the diabetic animals. There is evidence that *C. comatus* exhibits an antiobesity effect and plays a role in adipogenesis. Results from the differentiation of preadipocytes into adipocytes indicate that *C. comatus* extract inhibits intracellular triglyceride accumulation in 3T3-L1 adipocytes, resulting in a significant reduction in triglyceride content. The MTT (3-[4,5-dimethylthiazol-2-yl]-2,5 diphenyl tetrazolium bromide) assay demonstrated that *C. comatus* extract did not affect the viability of 3T3-L1 adipocyte cells at high concentrations. Experimental assays revealed that *C. comatus* extract prevented adipocyte differentiation by exerting an antagonistic effect on PPARγ (peroxisome proliferator-activated receptor-gamma) compared to the control group [4,6]. In vivo experiments in obese high-fat-diet-fed rats showed that treatment with *C. comatus* extract significantly reduced 32% of the total serum triglycerides and 46% of the total serum cholesterol levels. Additionally, the group treated with extract had a significant increase in high-density lipoprotein levels compared to the control group [4]. Although the animal model was different from ours, there is a similarity in the outcome regarding blood lipid analysis; diabetic animals treated with *C. comatus* had lower concentrations of total triglycerides and higher HDL cholesterol. Similarly, in a study that dealt with the antidiabetic effect of comatin, a substance isolated from *C. comatus*, it was shown that this active principle not only has a favorable impact on glycemia but also leads to a lowering of the concentration of total triglycerides [7]. Furthermore, research using a different model of diabetes pointed out that polysaccharides from *C. comatus* significantly lower total cholesterol and total triglycerides while increasing HDL cholesterol [3].

Another explanation for the impact of *C. comatus* on lipids may stem from its nutrient composition. Firstly, *C. comatus* typically contains low fat levels while exhibiting a high concentration of proteins. Secondly, its high fiber content, primarily non-digestible carbohydrates, further enhances its nutritional profile. Despite their low-fat content, mushrooms, including *C. comatus*, boast a notable percentage of essential fatty acids, such as linoleic acid, vital for human nutrition. Additionally, *C. comatus* contains various other essential fatty acids, including lauric, myristic, palmitic, oleic, α-linoleic, arachidonic, eicosatrienoic, and eicosapentaenoic acids, among others. Furthermore, the balance between saturated and unsaturated fatty acids significantly influences lipid metabolism. Previous analysis revealed that *C. comatus* contains a higher proportion of unsaturated fats compared to saturated fats. Given its richness in linoleic, oleic, and ω-3 fatty acids, *C. comatus* could play a pivotal role in promoting positive outcomes in fat metabolism observed in laboratory animals [2,5,12].

Various models of diabetes in different animal species and strains have proven the antidiabetic potential of the *C. comatus* mushroom. Similar to the results of our previous study on Wistar rats with alloxan diabetes, a dose of 1.67 g/kg *C. comatus* did not lead to a significant difference in glycemia compared to the control that received a saline solution, with a note that the treatment lasted significantly shorter. However, in the present study, the highest dose of mushroom led to a statistically significant reduction in glycemia compared to the control treatment [9]. The antidiabetic potential of *C. comatus* mushroom was also investigated in Wistar rats with streptozotocin-induced diabetes. Three varying doses of the ethanol extract from the fruiting body were administered daily for 14 days. The results revealed a dose-dependent impact on blood glucose levels, with the highest extract dose demonstrating the most pronounced effect. By the end of the experiment, blood sugar levels decreased by nearly 30% compared to baseline values. Furthermore, in vivo levels of DPP-4 were significantly reduced in animals treated with the extract compared to those treated with saline, resembling levels observed in healthy animals [8]. In the previously mentioned study, Kunming mice were treated for 60 days with polysaccharides isolated from *C. comatus* in three doses. In addition to the observed antidiabetic effect of the applied treatment, a negative correlation between the applied dose and the blood sugar value of the animals was also shown, so the highest dose achieved the greatest effect. Moreover, it is possible that by regulating carbohydrate metabolism, the treatment led to a positive impact on lipid values [3].

Looking at the results of the histological analysis comprehensively, it can be said that the treatment with mushroom did not lead to visible toxic effects on the pancreatic tissue. In contrast, it improved the regeneration of the beta fraction of Langerhans islet cells destroyed by alloxan (Figure 4). Although there is evidence that the pancreas regenerates itself after damage caused by substances such as streptozotocin and alloxan, this recovery is thought to be temporary. Namely, it has been shown that after self-recovery at week 2, there is a continuous blood glucose rise in the following weeks [15], evident from the glycemia results of the AloCON group here (Table 6). In the present research, the 42-day treatment with *C. comatus* contributed to the recovery of diabetic animals, initially manifested by the relative normalization of glycemia and histological parameters of morphometric analysis. In our earlier research, we already pointed out the protective potential of the *C. comatus* mushroom. Namely, a qualitative analysis of pancreatic tissue showed that the islets of Langerhans of animals treated for seven days with *C. comatus* were less damaged compared to the those of the control [9]. Furthermore, in a recent study with streptozotocin-induced diabetic Wistar rats, histological analysis proved the protective effect of *C. comatus*. Namely, classical H/E staining showed that with an increase in the dose of mushroom, there is a decrease in necrosis and inflammation and a dominance of normal pancreatic tissue [8]. The regenerative effect of *C. comatus* was indicated by Nowakowski et al. in their review paper, where the authors said that treatment with vanadium-enriched mushroom leads to histologically confirmed recovery of pancreatic beta cells in mice [4]. The hypothesis that certain substances, including drugs registered for the treatment of type 2 diabetes, can lead to the recovery of the endocrine part of the pancreas has been proven in several preclinical studies. Common to these studies is a methodology where the numerical and volume density of Langerhans islets are evaluated with the help of stereology and immunohistochemistry. Thus, in a study on Wistar rats with streptozotocin-induced diabetes, the influence of sinapic acid and ellagic acid, phenolic acids considered to have antidiabetic effects, was evaluated. Treatment of rats with a combination of these two substances led to an increase in the numerical density and volume of pancreatic beta cells, which positively affected blood sugar levels [13]. Additionally, the regenerative effect of semaglutide was demonstrated in research on C57BL/6 mice. Semaglutide led to an increase in cell proliferation and alpha and beta remodeling, which was accompanied by a decrease in pro-inflammatory markers and an improvement in the function of peroxisome proliferator-activated receptor (PPAR)-gamma and, ultimately, glycemic regulation [17]. A potential explanation for the success of the previously described treatments lies in the plasticity of pancreatic tissue. Namely, modern research indicates that adaptation of the pancreas to stress conditions, i.e., damage to beta cells, partly implies cellular trans-differentiation, respectively changing the role of other cells of the islets of Langerhans. It was discovered that within one cell line of the islets of Langerhans, there are cells with mixed phenotypic characteristics, which, in conditions of a lack of cells in other lines, can partially take over their role. It is even described that extra-islet cells, such as epithelial acinar and ductal cells, but also hepatocytes and intestinal epithelial cells have the potential to produce insulin and undergo trans-differentiation. One of the studies indicated that pancreatic polypeptide (Ppy)-expressing γ-cells but also alpha and delta cells experience changes in gene expression when the number of beta cells is reduced, so they begin to produce insulin [19,20,21,22]. The plasticity of the endocrine part of the pancreas is a mechanism of adaptation to acute stress. In contrast, under conditions of chronic stress, it is depleted, which is evident in type 1 diabetes [18]. There is even evidence that within the plasticity of the pancreas in diabetes, there could be a change in the cellular identity of the exocrine part, in acinar and ductal cells, which can eventually lead to the appearance of pancreatic carcinoma [28].

There are limitations to the current study. Firstly, alloxan diabetes cannot be fully translated into human diabetes. Although alloxan causes selective damage to beta cells that should correspond to type I diabetes in humans, alloxan does so chemically by producing free radicals. In contrast, in human type I diabetes, the damage is immune-mediated. Regardless, alloxan diabetes is still widely represented in research on potentially antidiabetic substances. Secondly, only one batch of *C. comatus* was used in the in vitro tests, and we saw the justification for that in that the same batch was used for the in vivo study. Additionally, in the in vitro studies of the antidiabetic effect, we did not compare the extracts obtained by different extraction methods because, as we stated earlier in the discussion, according to the results of our earlier studies, we expected that a higher content of phenolic compounds would contribute to a better antidiabetic effect. Finally, caution is necessary in interpreting the regenerative potential of all substances mentioned in the discussion, including *C. comatus* studied here. Thus, a more detailed examination of markers of pancreatic tissue plasticity, cellular dedifferentiation, and trans-differentiation is needed, which can undoubtedly be the direction of our future research on diabetes models.

## 4. Materials and Methods

### 4.1. Mushroom Samples and Extraction Procedure

The experiments utilized a *Coprinus comatus* (O. F. Müll.) Pers., prepared as a 100% powder from whole, cultivated mushrooms that were finely minced and lyophilized. This product is obtainable as a food supplement in Serbian pharmacies. Mushroom samples employed in the experiments were kept in desiccators and safeguarded from light exposure.

The extract was prepared in a dilution of 1:10 w:w (the ratio of dry matter of *C. comatus* mushroom to boiling water). Water was selected as the extraction solvent based on findings by Tešanović et al., who demonstrated that aqueous extraction of *Coprinus comatus* yields a favorable phenolic profile, with high content of chlorogenic acid [11]. The extraction was carried out using the standard method for 1 h at 80 °C in a water bath, with control by a thermometer immersed in the water to keep the temperature constant. After an hour, the mixture was filtered with the help of filter paper, and the filtrate was retained and then transferred to a measuring vessel and frozen at −20 °C until lyophilization. Lyophilization was performed using a Christ Alpha 1-2 LD Freeze Dryer (Osterode am Harz, Germany) at −70 °C. After 24 h, when the lyophilization was completed and the moisture was extracted from the sample, the mass of the vial with the sample was measured to obtain the exact mass of the lyophilized extract, making the final extraction yield 14.7%. Before each test, the extract was reconstituted according to the methods described below.

### 4.2. Chemicals

Formic acid, acetic acid, water high-performance liquid chromatography (HPLC) grade, ammonium acetate, methanol, dimethyl sulfoxide (DMSO), gallic acid, caffeic acid, trans-cinnamic acid, p-coumaric acid, chlorogenic acid, rosmarinic acid, ferulic acid, quercetin, rutin, quercitrin, potassium phosphate, reduced glutathione, Starch azure, porcine-origin α-amylase, α-glucosidase from Saccharomyces cerevisiae, p-nitrophenyl-α-D-glycoside, dipeptidyl peptidase (DPP)-4 enzyme, Gly-Pro-p-nitroanilide, and alloxan monohydrate were purchased from Merck (Darmstadt, Germany). Acarbose and urethane were obtained from Thermo Fisher Scientific (Waltham, MA, USA) and sitagliptin phosphate monohydrate from J&K Scientific (San Jose, CA, USA). All the other chemicals were of analytical grade and obtained from conventional sources.

### 4.3. HPLC Analysis of Phenolics

The examined extract underwent chemical characterization, and the selected compounds were quantified using a validated HPLC method [32]. Briefly, phenolic compounds were separated utilizing an Agilent Technologies 1100 liquid chromatographer equipped with a diode array detector (Agilent Technologies, Santa Clara, CA, USA). Separation occurred on a reversed-phase Nucleosil C18 column (250 mm × 4.6 mm, 5 μm particle size; Agilent Technologies) under controlled conditions. The mobile phase, consisting of aqueous formic acid with ammonium acetate (solvent A) and methanol (solvent B), was delivered in a gradient mode. The injection volume and run time were standardized, and quantification was conducted using chemical standards of phenolics. Data analysis was performed using Agilent OpenLAB Control Panel v.A.01.05. Results were reported as micrograms per kilogram of dry extract.

### 4.4. In Silico Docking Study

The three-dimensional crystal structures of human dipeptidyl peptidase-4 (DPP4) in a complex with sitagliptin (PDB code: 1X70), human lysosomal alpha-glucosidase in complex with acarbose (PDB code: 5NN8), and human pancreatic alpha-amylase in complex with acarbose (PDB code: 3BAJ), which have been determined by X-ray diffraction at resolutions of 2.10 Å, 2.45 Å, and 2.1 Å, respectively, were downloaded from the Protein Data Bank. Structures of ligands (cinnamic acid, p-coumaric acid, caffeic acid, and chlorogenic acid) in mol2 format were retrieved from the freely available SANCDB database https://sancdb.rubi.ru.ac.za/ (accessed on 15 June 2023). Molecular docking analysis was performed using Molegro Virtual Docker (MVD) software, version 6.0, according to the previously published method [33]. The docking scores and H bond scores were compared with the standard compounds (sitagliptin for 1X70, acarbose for 5NN8, and 3BAJ).

### 4.5. Inhibition of α-Amylase Activity

The extract’s ability to inhibit α-amylase was assessed following the protocol by [34]. Briefly, reaction mixtures comprising Starch azure^®^, different extract concentrations, and porcine-origin α-amylase were prepared. Controls lacked the extract. After 10 min of incubation, acetic acid was added to halt the reaction, followed by centrifugation and absorbance measurement at 595 nm wavelength using an Agilent UV/Vis 8453 spectrophotometer. Acarbose served as the positive control, and all measurements were conducted in triplicate. The percentage of inhibition was calculated compared to the absorbance of reaction mixtures containing no extract (100% inhibition).

### 4.6. Inhibition of α-Glucosidase Activity

The ability of the tested extract to inhibit α-glucosidase activity was determined following the protocol by Kladar et al., 2017 [35]. The reaction mixtures contained potassium phosphate buffer (pH = 6.8), glutathione solution (reduced form), an α-glucosidase enzyme isolated from *Saccharomyces cerevisiae* (final enzyme activity in the mixture was 7.6 U/L), and p-nitrophenyl- α-D-glycoside, which was used as a substrate. After adding the tested extracts in two different concentrations and incubating at 37 °C for 20 min, the reaction was stopped with Na_2_CO_3_. The absorbances of the resulting solutions were measured at 405 nm wavelength using an Agilent UV/Vis 8453 spectrophotometer. Acarbose was used as a positive control, and all measurements were performed in triplicate. The reaction mixture without the tested extract was considered to have 100% enzyme activity.

### 4.7. Inhibition of Dipeptidyl Peptidase (DPP)-4 Activity

The in vitro DPP-4 inhibition test involved three sets of test tubes, each performed in triplicate. Group I acted as the DPP-4 negative control, Group II as the DPP-4 + sitagliptin (positive control), and Group III as the DPP-4 + extract (test group). Sitagliptin phosphate monohydrate and the extract were dissolved in dimethyl sulfoxide and diluted with Tris buffer to a concentration of 10 mM/mL. DPP-4 human enzyme, with a final activity of 0.05 U/mL, was added to the assay mixtures, followed by incubation at 37 °C for 20 min. Subsequently, the chromogenic substrate Gly-Pro-p-nitroanilide was added, and substrate hydrolysis was monitored at 405 nm using an Agilent UV/Vis 8453 spectrophotometer. The activity was quantified as ΔA405 nm/min [14]. The reaction mixture without the tested extract was considered to have 100% enzyme activity.

### 4.8. Laboratory Animals

White Wistar laboratory rats, both male and female (three rats of each sex housed separately in cages for each group), weighing between 250 and 350 g and aged three months, were sourced from local animal facilities (Military Medical Academy, Belgrade, Serbia). The rats were kept in the UniProtect airflow cabinet (Ehret GmbH, Emmendingen, Germany) in standard Plexiglas cages, maintaining a constant room temperature of 22 ± 1 °C, humidity of 55% ± 1.5%, and a standard 12-h day/night cycle. Throughout the experiment, the rats had ad libitum access to tap water and standard pelleted laboratory rodent feed (Veterinary Institute Subotica, Serbia). Their health was monitored daily. The experimental procedures were conducted per the European Directive (2010/63/EU) for animal experiments, and they were reviewed and approved by the Ethics Committee for Protection and Welfare of Experimental Animals at the University of Novi Sad, Serbia (Approval No. III-2011-07).

### 4.9. Experimental Procedures

The antidiabetic properties of *C. comatus* were investigated using a rat model of alloxan-induced diabetes. After a week of adaptation before the experiments, 48 rats were randomly divided into eight groups (n = 6) as follows:

CON group—rats received saline per os (p.o.) (2 mL/kg/day) for 42 days;

C1 group—rats received *C. comatus* p.o. (0.835 g/kg/day) for 42 days;

C2 group—rats received *C. comatus* p.o. (1.67 g/kg/day) for 42 days;

C3 group—rats received *C. comatus* p.o. (3.34 g/kg/day) for 42 days;

AloCON group—diabetic rats received saline p.o. (2 mL/kg/day) for 42 days;

AloC1 group—diabetic rats received *C. comatus* p.o. (0.835 g/kg/day) for 42 days;

AloC2 group—diabetic rats received *C. comatus* p.o. (1.67 g/kg/day) for 42 days;

AloC3 group—diabetic rats received *C. comatus* p.o. (3.34 g/kg/day) for 42 days.

All animals were weighed before the administration of the first dose of the tested substances (BW start) then weekly until the final measurement on the last day of the experiment (BW end). *Coprinus comatus* treatment groups were administered an aqueous suspension of mushroom orally in three different doses using an intragastric probe for rats. An administered dose of 3.34 g/kg correlates approximately to one standard serving of 30 g dry-weight mushroom for the average person (60 kg), calculated using the Food and Drug Administration’s Human Equivalent Dose formula. The other two mushroom doses were half and a quarter of the previously mentioned dose. Control groups received an orally administered 2 mL/kg dose of isotonic saline using an intragastric probe for rats. On the last day of the experiment, animals were put under urethane anesthesia (5 mL/kg i.p. of 25% solution) and exsanguinated with intracardial punction. The blood was collected and centrifuged for 10 min at 3000 rpm and 4 °C to separate serum. The pancreas was also rapidly collected, rinsed with ice-cold saline, and transferred to 10% buffered formalin for histopathological examination.

The concentration of glucose in capillary blood, taken from the tail vein of rats, was determined with commercial kits on the Accu-chek Active device (Roche, Basel, Switzerland). The blood glucose concentration of the experimental animals was measured around 10 AM, two hours after the first dose of mushroom or saline (BG start), and then every seven days until the end of the trial. The last blood glucose measurement was taken two hours after the last dose of mushroom or saline (BG end). To determine the effect of treatment on blood sugar values as precisely as possible, the area under the blood glucose concentration curve (BG AUC) during the observed period was calculated using the trapezoidal method. All glycemic measurements were considered to calculate the area under the curve.

In the normoglycemic animals (without alloxan), the oral glucose tolerance test (OGTT) was performed by administering the aqueous glucose solution at 3 g/kg per animal. The OGTT was carried out on the last day of the treatment, two hours after administering the last dose of saline or *C. comatus*. Before applying the aqueous glucose solution, the starting point of glycemia was determined (BG end), and then, after 30 min, the glycemia value was measured again (BG OGTT).

Alloxan was used to induce permanent hyperglycemia in laboratory animals. Alloxan was dissolved in saline solution immediately before the application and was applied in a dose of 150 mg/kg intraperitoneally (i.p.) once [15]. Before the application of alloxan, starting glycemia was measured (BG before). After 48 h from the application of alloxan, blood was sampled from the tail vein to confirm the diagnosis of diabetes (BG alo). Animals that had glycemia higher than 15 mmol/L were included in the study. All the other animals were euthanized according to the humane endpoint ethical principle.

### 4.10. Biochemical Parameters Analysis

The serum was used to analyze total triglycerides (TG), total cholesterol (HOL), high-density lipoprotein cholesterol (HDL), and low-density lipoprotein cholesterol (LDL). The serum concentrations of these lipids were measured according to clinical biochemistry standard spectrophotometric methods on a fully automated analyzer, Olympus AU400 (Hamburg, Germany).

### 4.11. Immunohistochemical Analysis

After a complete autopsy, pieces of the splenic region of the pancreas were separated and fixed in Boujen’s fixative at 4 °C for 24 h. The tissue was then dehydrated, embedded in paraffin, and cut at a thickness of 5 μm for histological sections. Two adjacent tissue sections were taken from each mold, one for each immunohistochemical method. In the immunohistochemical tissue staining were the primary antibodies insulin Ab-6 (Lab Vision—Thermo Scientific, Waltham, MA, USA) and glucagon Ab-1 (Lab Vision—Thermo Scientific) and the visualization system UltraVision LP Detection System HRP Polymer & AEC Chromogen (Lab Vision—Thermo Scientific) of the same manufacturer. After rehydrating the sections (“bringing them to water”), they were subjected to a retrieval reaction, which involved heating the sections in citrate buffer (pH 6.0) at 850 W in a microwave oven for 20 min, followed by cooling the preparation for 30 min. After cooling, the sections were washed four times in buffer (TBS, pH7.4) and then, to eliminate non-specific background staining, incubated for 10–15 min in Hydrogen Peroxide Block (Lab Vision, TA-015-HP, Fremont, CA, USA), washed four times in buffer, and incubated for five minutes in Ultra V Block (Lab Vision TA-015-UB). After washing in the buffer, the appropriate primary antibody (insulin—ready to use, glucagon—diluted 1:100) was applied for 30 min at room temperature. The sections were then subjected to Primary Antibody Enhancer (Lab Vision, TL-015-PB) treatment for 20 min. After washing with buffer, the sections were subjected to HRP Polymer (Lab Vision, TL-015-PH) for thirty minutes. Visualization was performed using AEC Chromogen (Lab Vision, TA-015-SA). Basophilic structures were stained and contrasted using Mayer’s hematoxylin, and the preparations were mounted using a water-based adhesive (Bio-Optica, Milan, Italy). The preparations were analyzed on a Leica DMLB (Leica, Wetzlar, Germany) and photographed with a LeicaDC 100 (Leica, Germany) camera.

### 4.12. Morphometrical Analyses

A total of 96 adjacent sections (2 per animal sample) of the splenic region were used for morphometric analyses, 48 for insulin and 48 for glucagon. In each animal sample, 15–20 insulin-stained islets of Langerhans and the corresponding glucagon-stained islets in the adjacent section were examined at a magnification of ×400 or ×630 then photographed and analyzed using morphometrical formulas [17,36]. Islets were examined to estimate average values of numerical density of alpha and beta cells (N_Acα_ and N_Acβ_); average volumes of alpha and beta islet fractions (V_iα_ and V_iβ_); average values of the corrected mean nuclear volume (V_nα_ and V_nβ_); volume density of the endocrine and exocrine parts of the pancreas (V_vend_ and V_vegzo_); and the average number of alpha and beta cells per islet (N_α_ and N_β_). Free ImageJ software version 1.54f (National Institutes of Health, Bethesda, MD, USA) was used for image processing (Supplementary Materials S2).

### 4.13. Statistical Analysis

Statistical analysis was performed using IBM SPSS statistical software, version 26.0 (IBM Corp., Armonk, NY, USA). Data were reported as the mean ± standard deviation (s.d.) or standard error of the mean (SEM). For all statistical comparisons, normoglycemic and diabetic groups were analyzed separately using appropriate tests for multiple comparisons, depending on data distribution and study design. An independent samples T-test was used to compare the means of the two groups. A one-way analysis of variance (ANOVA) or Kruskal–Wallis test was employed to compare more than two experimental groups. Post hoc testing for ANOVA was performed using Tukey’s test. Mann–Whitney U test with or without Bonferroni correction was used for post hoc testing in the Kruskal–Wallis analysis. Correlation analysis was performed using Pearson’s coefficient of linear correlation. Assessment of dose-dependent response was carried out by linear trend analysis. The difference between groups was considered statistically significant for a *p*-value less than 0.05 (*p* < 0.05).

## 5. Conclusions

This study demonstrated that a 42-day treatment restored both the number and function of insulin-secreting cells, as indicated by biochemical and histological analyses. Given these findings, further investigation should include in-depth spectrophotometric analysis of other potential bioactive compounds, beyond just phenolic compounds, in *C. comatus*, followed by in silico and in vitro studies, and ultimately validation in an in vivo model.

## Figures and Tables

**Table 1:** Contents of selected phenolic compounds in *C. comatus* extract.

Class of Phenols	Phenolic Compound	Content [µg/g]
Phenolic acids	Gallic acid	ND
	Caffeic acid	12.88 ± 0.64
	trans-Cinnamic acid	ND
	p-Coumaric acid	6.51 ± 0.65
	Chlorogenic acid	25.48 ± 1.27
	Rosmarinic acid	ND
	Ferulic acid	ND
Flavonoids	Quercetin	ND
	Rutin	ND
	Quercitrin	ND

ND—not detected; values represent mean ± standard deviation of triplicate analysis.

**Table 2:** Molecular docking study of selected phenolics from *C. comatus*.

	1X70 (DPP4)		5NN8 (Alpha-Glucosidase)		3BAJ (Alpha-Amylase)	
Compound	MolDock Score (kcal/mol)	H Bond (kcal/mol)	MolDock Score (kcal/mol)	H Bond (kcal/mol)	MolDock Score (kcal/mol)	H Bond (kcal/mol)
Sitagliptin/acarbose	−152.99	−2.16	−88.60	−9.63	−157.24	−25.16
p-coumaric acid	−90.07	−4.53	−63.21	−4.80	−70.92	−3.98
caffeic acid	−80.72	−9.71	−67.35	−7.97	−81.29	−9.02
chlorogenic acid	−130.90	−17.99	−88.40	−9.32	−98.22	−11.45

**Table 3:** Enzyme inhibition activity of *C. comatus* extract, acarbose, and sitagliptin expressed as IC_50_ (50% inhibition) values (µg/mL).

Samples	Enzymes		
	α-Amylase	α-Glucosidase	DPP4
*C. comatus*	13,629.74 ± 1026.67	757.48 ± 84.45	1650.31 ± 145.29
Acarbose	4.93 ± 0.46	42.87 ± 3.96	n.a.
Sitagliptin	n.a.	n.a.	0.02 ± 0.001

n.a.—not applicable.

**Table 4:** Body weights of rats treated for 42 days with *C. comatus* or saline (mean ± SEM; n = 6; BW start—body weight before treatment; BW end—body weight on the last day of treatment; ΔBW—change in body weight).

Group	BW Start	BW End	ΔBW
CON	245.17 ± 5.64	316.33 ± 5.14	71.17 ± 8.35
C1	258.67 ± 11.86	349.50 ± 18.84	90.83 ± 7.56
C2	249.67 ± 8.44	332.17 ± 19.83	82.50 ± 14.22
C3	265.50 ± 12.71	342.17 ± 19.11	76.67 ± 7.31
AloCON	254.17 ± 4.28	296.83 ± 7.05	42.67 ± 8.48
AloC1	263.17 ± 11.58	355.67 ± 26.24	92.50 ± 14.89
AloC2	261.83 ± 4.62	318.50 ± 18.05	68.60 ± 17.09
AloC3	263.67 ± 10.69	337.50 ± 26.60	73.83 ± 16.40

**Table 5:** Blood glucose values in normoglycemic animals after 42 days of treatment with *C. comatus* or saline (mean ± SEM; n = 6).

Group	BG Start	BG End	BG AUC	BG OGTT	ΔBG
CON	7.93 ± 0.12	6.72 ± 0.16	281.74 ± 8.20	12.65 ± 0.41	5.83 ± 0.32
C1	7.80 ± 0.19	6.28 ± 0.23	264.60 ± 5.50	13.53 ± 0.53	6.43 ± 0.49
C2	7.55 ± 0.13	5.48 ± 0.20 ^#^	287.64 ± 3.45	12.23 ± 0.74	5.60 ± 0.62
C3	7.97 ± 0.14	6.30 ± 0.11	277.73 ± 4.92	14.20 ± 0.42	7.15 ± 0.39

# *p* < 0.05 vs. CON; BG start—blood glucose before the treatment; BG end—blood glucose on the last day of the experiment; AUC—area under the curve; BG OGTT—blood glucose after oral glucose tolerance test; ΔBG—change in blood glucose level between BG OGTT and BG end.

**Table 6:** Blood glucose values in diabetic animals after 42 days of treatment with *C. comatus* or saline (mean ± SEM; n = 6).

Group	BG Before	BG Alo	BG End ^1^	BG AUC ^1^	ΔBG ^1^
AloCON	7.62 ± 0.53	32.02 ± 0.44	24.67 ± 3.53	1116.79 ± 72.42	7.35 ± 3.22
AloC1	8.20 ± 0.35	32.53 ± 0.26	24.37 ± 0.60	1081.85 ± 35.29	8.17 ± 0.51
AloC2	7.83 ± 0.30	30.35 ± 1.44	16.98 ± 3.67	874.65 ± 170.41	13.37 ± 2.31
AloC3	7.00 ± 0.13	27.86 ± 2.38	11.30 ± 2.21 *	681.96 ± 158.69	16.45 ± 2.10 *

* *p* < 0.05 vs. AloCON; BG before—blood glucose before the application of alloxan; BG alo—blood glucose 48 h after alloxan application; BG end—blood glucose on the last day of the experiment; AUC—area under curve; ΔBG—change in blood glucose level between BG alo and BG end; 1—a significant correlation *p* < 0.01.

## Data Availability

All data are available from the corresponding author upon reasonable request.

## Supplementary Materials

The following supporting information can be downloaded at: https://www.mdpi.com/article/10.3390/molecules30112261/s1, Supplementary Materials S1: Chromatograms of selected phenolic compounds; Supplementary Materials S2: Morphometrical analyses—example of Free Image Tool 3.0 and ImageJ software processing.
