# Supplementary material for: In Vivo Regenerative Potential of Coprinus comatus in Pancreatic Tissue After Acute Stress with Chronic Consequences
Source: Molecules. 2025 May 22;30(11):2261. doi: 10.3390/molecules30112261 (PMC12155635; doi:10.3390/molecules30112261)
Supplement: Supplementary file 1 [file molecules-30-02261-s001.zip › Supplementary Material S2.pdf]

Morphometrical analyses – example of Free Image Tool 3.0 and ImageJ software processing

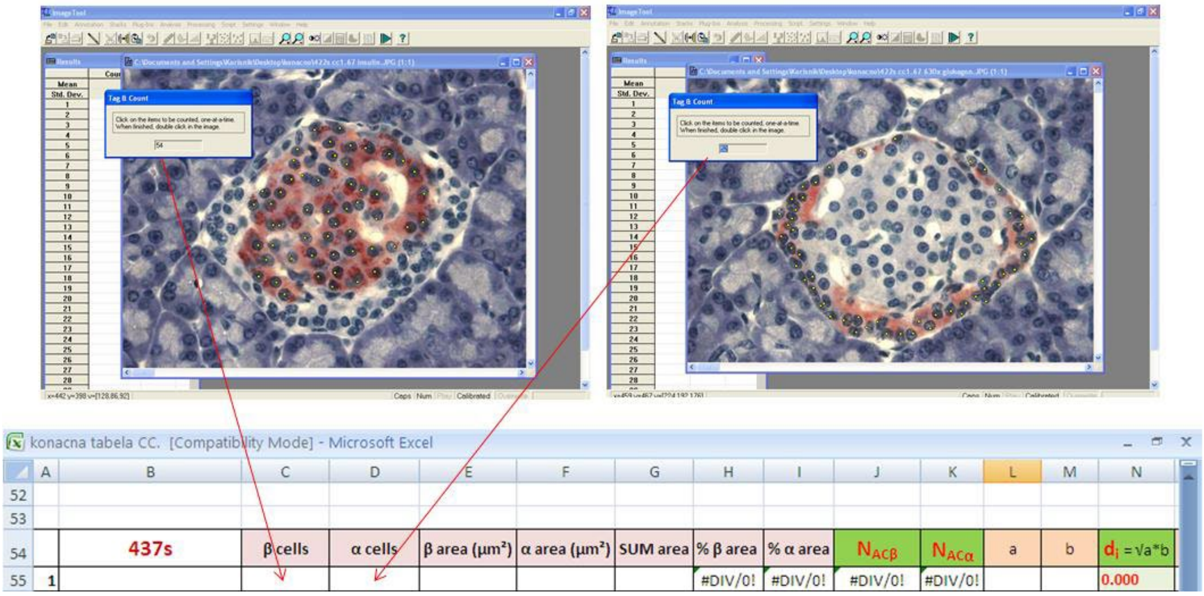

- Measuring of alfa and beta fraction area

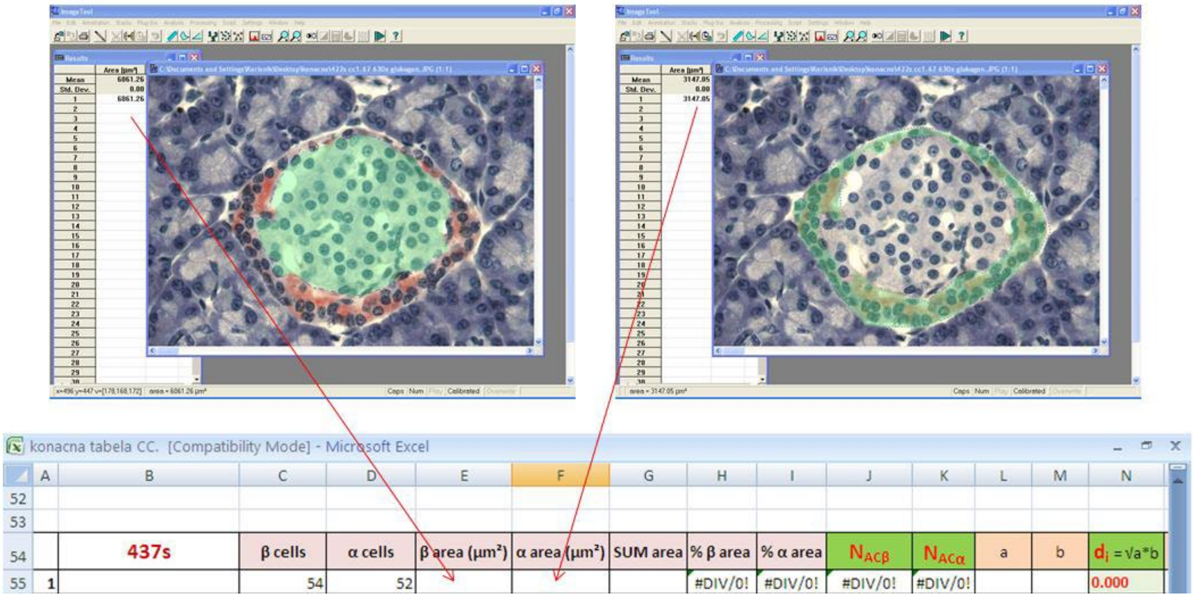

- Measuring of Langerhans islet diameter

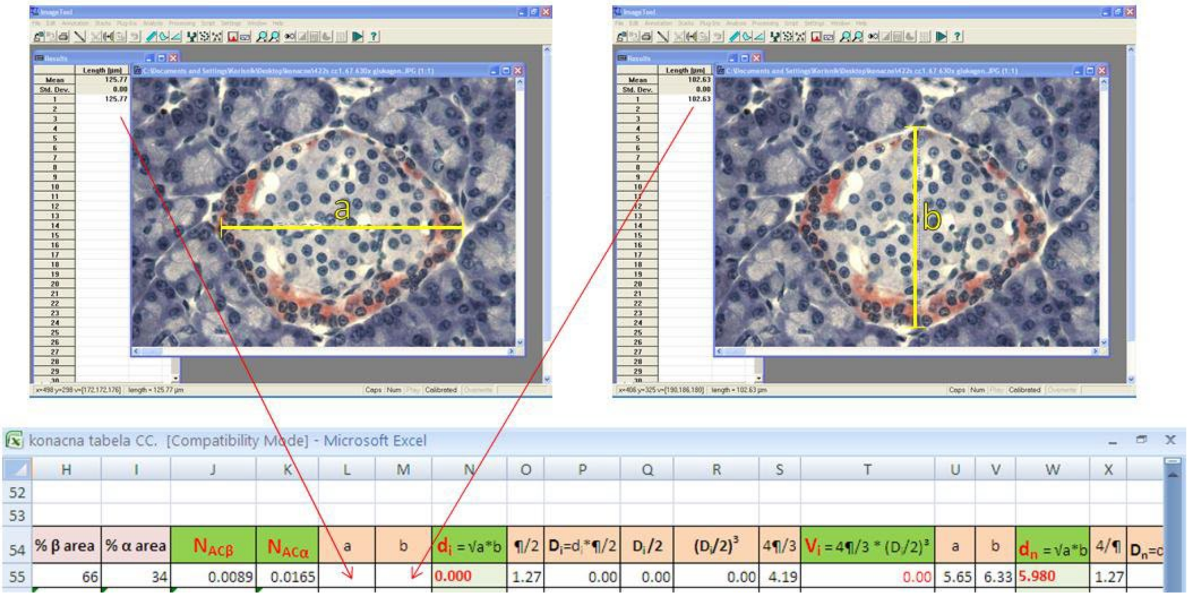

konacna tabela CC. [Compatibility Mode] - Microsoft Excel

	H	I	J	K	L	M	N	O	P	Q	R	S	T	U	V	W	X	
52																		
53																		
54	% β area	% α area	$N_{AC\beta}$	$N_{AC\alpha}$	a	b	$d_i = \sqrt{a*b}$	¶/2	$D_i = d_i * ¶/2$	$D_i/2$	$(D_i/2)^3$	4¶/3	$V_i = 4¶/3 * (D_i/2)^3$	a	b	$d_n = \sqrt{a*b}$	4/¶	D_n=c
55	66	34	0.0089	0.0165			0.000	1.27	0.00	0.00	0.00	4.19	0.00	5.65	6.33	5.980	1.27	

- Measuring of nuclear diameter

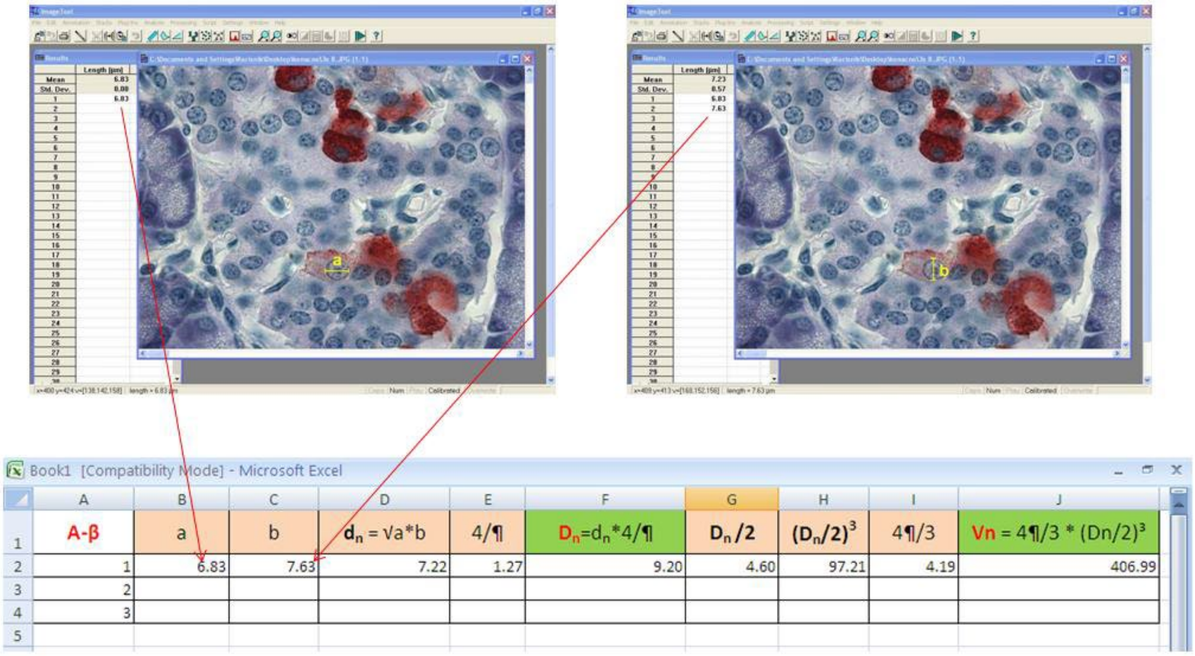

Book1 [Compatibility Mode] - Microsoft Excel

	A	B	C	D	E	F	G	H	I	J
1	A-β	a	b	$d_n = \sqrt{a*b}$	4/¶	$D_n = d_n * 4/¶$	$D_n/2$	$(D_n/2)^3$	4¶/3	$Vn = 4¶/3 * (Dn/2)^3$
2	1	6.83	7.63	7.22	1.27	9.20	4.60	97.21	4.19	406.99
3	2									
4	3									
5										
