# Supplementary figures and images for: In Vivo Regenerative Potential of Coprinus comatus in Pancreatic Tissue After Acute Stress with Chronic Consequences
Source: Molecules. 2025 May 22;30(11):2261. doi: 10.3390/molecules30112261 (PMC12155635; doi:10.3390/molecules30112261)

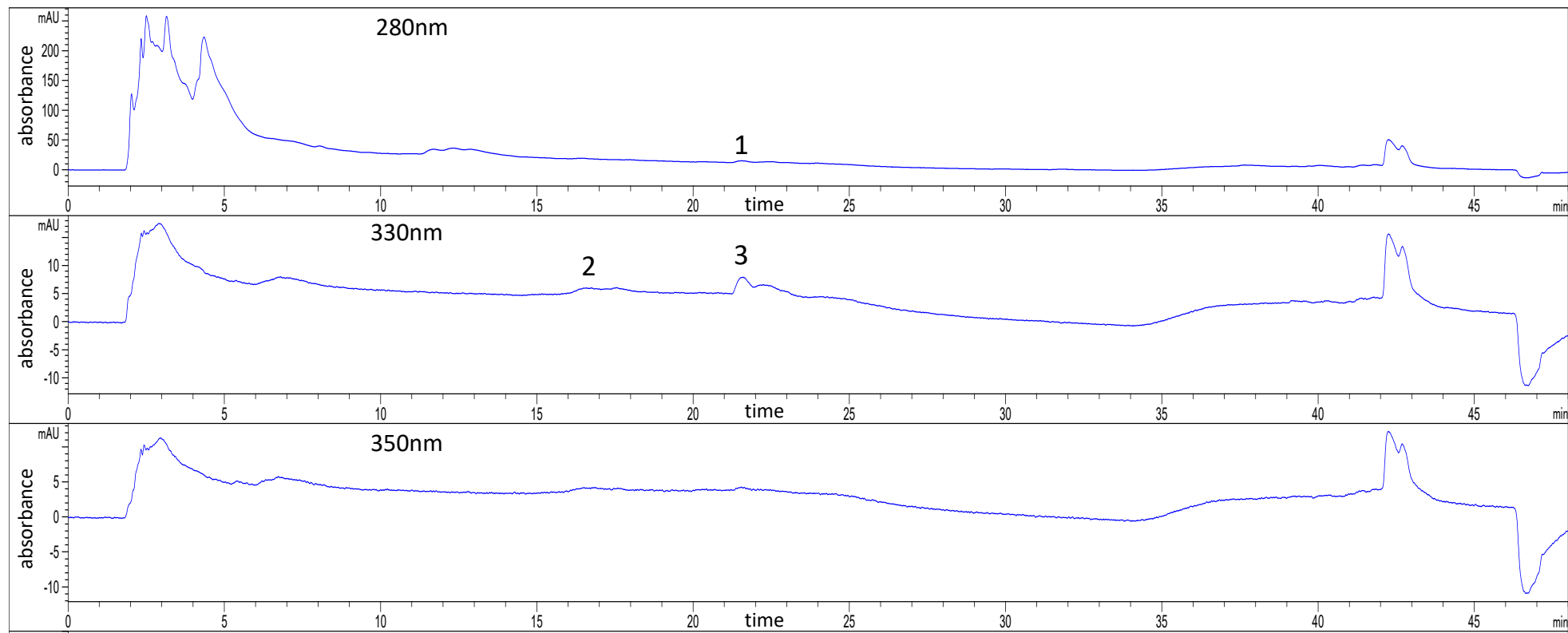

1 – caffeic acid  
2 – p-coumaric acid  
3 – chlorogenic acid

Supplement: Supplementary file 1 [file molecules-30-02261-s001.zip › Supplementary Material S1.pdf]
